# Clinical picture of early-infancy PTH resistance syndromes: is it time to improve diagnostic criteria?

**DOI:** 10.1210/clinem/dgag132

**Published:** 2026-04-08

**Authors:** Giulia Del Sindaco, Jugurtha Berkenou, Angela Pagnano, Christelle Audrain, Emanuele Ferrante, Anya Rothenbuhler, Agnès Linglart, Giovanna Mantovani

**Affiliations:** Endocrinology Unit, Fondazione IRCCS Ca’ Granda – Ospedale Maggiore Policlinico, Milan 20122, Italy; AP-HP, Service d’Endocrinologie et Diabète de l’enfant, Hôpital Bicêtre Paris-Saclay, Le Kremlin-Bicêtre 94270, France; AP-HP, Centre de Référence des Maladies Rares du Métabolisme du Calcium et du Phosphate, Filière OSCAR, ERN BOND, ERN for Rare Endocrine Disorders, Plateforme D’expertise des Maladies Rares de Paris Saclay, Le Kremlin-Bicêtre 94270, France; Endocrinology Unit, Fondazione IRCCS Ca’ Granda – Ospedale Maggiore Policlinico, Milan 20122, Italy; Department of Clinical Sciences and Community Health, Dipartimento di Eccellenza 2023-2027, University of Milan, Milan 20122, Italy; AP-HP, Service d’Endocrinologie et Diabète de l’enfant, Hôpital Bicêtre Paris-Saclay, Le Kremlin-Bicêtre 94270, France; AP-HP, Centre de Référence des Maladies Rares du Métabolisme du Calcium et du Phosphate, Filière OSCAR, ERN BOND, ERN for Rare Endocrine Disorders, Plateforme D’expertise des Maladies Rares de Paris Saclay, Le Kremlin-Bicêtre 94270, France; Endocrinology Unit, Fondazione IRCCS Ca’ Granda – Ospedale Maggiore Policlinico, Milan 20122, Italy; AP-HP, Service d’Endocrinologie et Diabète de l’enfant, Hôpital Bicêtre Paris-Saclay, Le Kremlin-Bicêtre 94270, France; AP-HP, Centre de Référence des Maladies Rares du Métabolisme du Calcium et du Phosphate, Filière OSCAR, ERN BOND, ERN for Rare Endocrine Disorders, Plateforme D’expertise des Maladies Rares de Paris Saclay, Le Kremlin-Bicêtre 94270, France; AP-HP, Service d’Endocrinologie et Diabète de l’enfant, Hôpital Bicêtre Paris-Saclay, Le Kremlin-Bicêtre 94270, France; AP-HP, Centre de Référence des Maladies Rares du Métabolisme du Calcium et du Phosphate, Filière OSCAR, ERN BOND, ERN for Rare Endocrine Disorders, Plateforme D’expertise des Maladies Rares de Paris Saclay, Le Kremlin-Bicêtre 94270, France; Université Paris Saclay, INSERM UMR 1185, AP-HP, Service d'Endocrinologie et diabète de l'enfant, Hôpital Bicêtre Paris Saclay, Le Kremlin-Bicêtre 94270, France; Endocrinology Unit, Fondazione IRCCS Ca’ Granda – Ospedale Maggiore Policlinico, Milan 20122, Italy; Department of Clinical Sciences and Community Health, Dipartimento di Eccellenza 2023-2027, University of Milan, Milan 20122, Italy

**Keywords:** pseudohypoparathyroidism, newborns, infancy, diagnostic criteria

## Abstract

**Context:**

Clinical presentation of inactivating parathyroid hormone (PTH)/PTH–related protein (PTHrP) signaling disorders (iPPSDs, historically pseudohypoparathyroidism [PHP]) exhibits pronounced age-dependence. Indeed, main features, including PTH resistance and brachydactyly (BD), develop during late childhood, while other features (ectopic ossifications, obesity, and hypothyroidism) are most prevalent in toddlers. The latter are included among minor diagnostic criteria; therefore, a substantial diagnostic delay has been reported.

**Objective:**

The aim of this work is to describe the early natural history of a large cohort of iPPSD/PHP patients to improve diagnosis, with the final goal of proposing new diagnostic criteria for early infancy and reducing diagnostic delay.

**Methods:**

We included 117 patients regularly followed up at 2 European endocrinology tertiary centers, and we retrospectively collected data on the age of onset of main clinical and hormonal features.

**Results:**

In our cohort the median age at diagnosis was 5.9 years. Age of onset of PTH resistance and BD, major criteria for diagnosis, was significantly different from that of both thyrotropin (TSH) resistance and obesity (median age 6.1, 5.8, 1.85, and 2 years, respectively). Minor diagnostic criteria were more represented than major criteria in children before age 2 years (*P* = .002). Indeed, in 64% of patients before age 2 years none of the major criteria were observed; conversely, 71% had already developed at least one minor criterion. In particular, 20% had developed TSH resistance and obesity.

**Conclusion:**

The clinical picture of iPPSD/PHP in early infancy differs from that of mid-late infancy and adults, thus current diagnostic criteria may not be appropriate for children. We suggest that the combination of early-onset obesity and elevated TSH should raise suspicion and trigger genetic screening before age 2 years.

Parathyroid hormone (PTH) resistance syndromes include a large spectrum of disorders historically named pseudohypoparathyroidism (PHP) and related disorders and newly referred to as inactivating PTH/PTH–related protein (PTHrP) signaling disorders (iPPSDs) (1). This group of syndromes is characterized by physical findings, variably associated in each subtype, as well as hormonal and neurocognitive abnormalities (2).

Fuller Albright and colleagues first described PHP in 1942, as the first hormonal resistance to be characterized (3). Since then a remarkable clinical variability and/or atypical phenotypes have been observed, leading to the new classification and nomenclature, which is basically conceived around the pathophysiology and the underlying genetic defect (1). Indeed, all these disorders result from impairments in the cyclic adenosine monophosphate (cAMP)-mediated signal transduction pathway, particularly in the PTH/PTHrP signal transduction cascade. A better understanding of this pathway elucidated the pathophysiology of PTH resistance syndromes and therefore the genetic background. The most frequent subtypes of iPPSD/PHP are caused by de novo or autosomal dominantly inherited mutations or methylation defects within the *GNAS* complex locus, an imprinted locus encoding the α-subunit of the stimulatory G protein (Gsα) and several other transcripts (4, 5). Moreover, mutations in *PRKAR1A* and *PDE4D*, which are crucial for Gsα-cAMP-mediated signaling, have been found in patients affected by acrodysostosis type 1 and 2, iPPSD4 and iPPSD5, respectively (6, 7). In addition to *PRKAR1A* and *PDE4D* variants, *PDE3A* mutations have also been identified within the PTH1R/Gsα/Camp/PKA pathway, although they are associated with a distinct phenotype characterized by hypertension and brachydactyly (BD) type E (8).

A molecular cause can be identified in about 80% to 90% of patients with iPPSD/PHP (9, 10), but clinical and biochemical overlap is frequently described in these patients. Thus, in the absence of a molecular analysis the diagnostic classification and the understanding of the natural history can be challenging (5), adding more difficulties to the management of this group of disorders.

As for clinical presentation, iPPSD/PHP encompasses a heterogeneous group of diseases, highly variable in clinical features and disease severity, even among patients carrying the same genetic background (5). Clinical and biochemical features that show minimal or no overlap with other diseases have been defined as major criteria for the diagnosis of iPPSD/PHP (11), as suggested by international experts (1); these include PTH resistance, ectopic ossifications (EOs), and BD, the latter associated with at least one major or two minor features. Conversely, minor criteria are less specific to iPPSD/PHP and common to other clinical conditions, that is, thyrotropin (TSH) resistance; other hormone resistances such as growth hormone releasing hormone (GHRH), calcitonin, gonadotropins, glucagon, and adrenaline; motor and cognitive retardation or impairment; intrauterine and postnatal growth retardation; early-onset obesity and overweight; or facial dysmorphisms (1). Clinical presentation at diagnosis is highly variable in an age-dependent manner, as many of the features, including the 3 major criteria, develop during mid and late childhood (5). PTH resistance, the hallmark of these disorders, is usually absent at birth, and overt subsequent hypocalcemia is detected in most patients by age 7 to 8 years (11-13). Similarly, BD is absent in most patients during early infancy and becomes evident during puberty (1, 11, 14). On the contrary, other features such as TSH resistance or obesity/overweight, currently included among minor diagnostic criteria, are often already present during early infancy (15-17). Therefore, the clinical picture of early infancy iPPSD/PHP is quite different from and less specific than that of late childhood and adulthood, and in this scenario a considerable delay in diagnosis may be common (16, 18). Diagnostic delay in iPPSD/PHP may have several relevant implications. It can lead to severe complications, such as hypocalcemia-related seizures, and limit the opportunity for timely monitoring of growth and initiation of growth hormone (GH) replacement therapy. Moreover, delayed diagnosis may hamper appropriate genetic counseling for families and compromise the coordination of multidisciplinary care for affected patients.

The aim of the present work is to describe the early natural history of iPPSD/PHP by analyzing a large cohort of patients to improve the diagnostic work-up and reduce diagnostic delay in these patients, with the final goal to suggest new diagnostic criteria specific for early infancy.

## Materials and methods

### Patients

We collected data from 117 patients affected by iPPSD/PHP, regularly followed up at the Endocrinology Unit of Fondazione IRCCS Ca’ Granda Ospedale Maggiore Policlinico (Milan, Italy) and at the Department of Endocrinology and Diabetes for Children of Bicêtre Paris Saclay Hospital (Le Kremlin-Bicêtre, France). These patients were extracted from the same cohort of patients that we have recently studied focusing on the occurrence of neonatal complications (19), ruling out patients whose age of onset of main clinical and biochemical features could not be dated. Sixty-seven children and 50 adults were included in this study (67 females and 50 males, median age 16.5 years). All patients had a genetically confirmed diagnosis. Detailed information on genetic variants and inheritance patterns is provided in Supplementary Table S1 (20). Sixty-two patients (53%) were diagnosed with iPPSD2 (PHP1A), 21 (18%) with iPPSD3 (PHP1B), 11 (9.4%) with iPPSD4 (acrodysostosis type 1, mutation in *PRKAR1A*), 5 (4.3%) with iPPSD5 (acrodysostosis type 2, mutation in *PDE4D*), 3 (2.5%) with iPPSD1 (mutation in *PTH1R*), and 15 (12.8%) with iPPSDx (unknown mutation). The study was approved by the local ethical committee of Milan Area 2 (ID: 841, approval No. 15_2019bis) and was conducted in accordance with the Declaration of Helsinki. All patients or their legal guardians provided written informed consent prior to study participation.

### Methods

We retrospectively extracted from each medical record data about the age of onset or the age of diagnosis of the main clinical and biochemical features (major and minor diagnostic criteria).

We defined early infancy as the period before age 2 years, thus including infants (age 1 to <12 months) and toddlers (age 1 to ≤2 years).

Resistance to PTH was defined as the association of an increased serum level of PTH and elevated serum phosphate (>normal range for age: birth to 4 years, 1.3-2.2 mmol/L; 4-10 years, 1.2-1.7 mmol/L; 10-18 years, 1.1-1.8 mmol/L; and >18 years, 0.8-1.5 mmol/L) and/or low serum calcium (≤2.20 mmol/L) in the absence of vitamin D deficiency and chronic kidney disease. Serum 25-hydroxyvitamin D levels were assessed on an individual patient basis as part of the diagnostic biochemical evaluation; consequently, vitamin D deficiency was excluded or, when present, adequately supplemented in all patients before establishing the biochemical diagnosis of PTH resistance. The same applies to chronic kidney disease, which was ruled out in all cases based on standard clinical and biochemical evaluations.

Brachydactyly was described at physical examination or as radiological evidence of variable shortening of the metacarpals, occasionally accompanied by relatively shortened metatarsals. Ectopic ossifications were evaluated by careful clinical examination.

Resistance to TSH was defined as elevated serum levels of TSH with normal or slightly reduced levels of thyroid hormones. In some cases, we considered as the age of onset of hormonal resistances the time at which replacement therapies for PTH and TSH resistance were initiated, with active vitamin D analogues or levothyroxine, respectively. Regarding PTH resistance, treatment was generally initiated when PTH levels exceeded twice the upper limit of normal (ULN), regardless of the presence of hypocalcemia, in line with current recommendations (5).

In both cohorts (French and Italy), neonatal screening for congenital hypothyroidism, based on TSH measurement, was systematically performed according to national standards.

All biochemical measurements were performed in local certified laboratories using standardized and validated assays routinely adopted in clinical practice. For PTH measurements, values were also reported as ULN to account for differences among assays used across centers.

The presence of obesity was described according to the specific growth charts as weight > 3 SD before age 2 years and body mass index (BMI) > IOTF (International Obesity Task Force) 30 after 2 years, whereas overweight was defined as weight > 2 SD before age 2 years and BMI > IOTF 25 after 2 years. In children older than 2 years, the extended IOTF BMI cutoffs were used (21). We respectively used Italian or French growth charts or World Health Organization growth charts for children of other nationalities.

Neurocognitive impairment (NC) was not systematically diagnosed through standardized neuropsychological tests but was defined by the caring physician as global retardation of developmental milestones, psychomotor retardation, delayed speech, or the need for an assistant teacher and extra school help.

### Statistics

Quantitative variables describing patient characteristics, including age (years), age at diagnosis (years), and age of onset of main clinical features (years), were expressed as medians with interquartile range (IQR) or means ± SD according to their distribution. Qualitative data were described in numbers and percentages (%).

Normal distribution was verified using Kolomogorov-Smirnov test.

The Wilcoxon signed rank test was used to compare age of onset of main clinical and biochemical features of iPPSD/PHP, as the variables were not normally distributed. Categorical data were analyzed using the chi-square test or Fisher’s exact test if the expected value was < 5.

Kaplan-Meier curves were plotted for each major criterion, showing the proportion of patients remaining event-free over time.

*P* values < .05 were considered statistically significant. All statistical analysis, tables, and figures were created using SPSS version 27 (IBM), Prism version 7.0, and R software version 4.4.1.

## Results

In our cohort of patients, the mean age at diagnosis was 7.2 ± 6.7 years (median 5.9 years; IQR 1.8-11.0 years), being significantly lower in patients affected by iPPSD2/PHP1A (*P* < .001).

**Figure 1:**
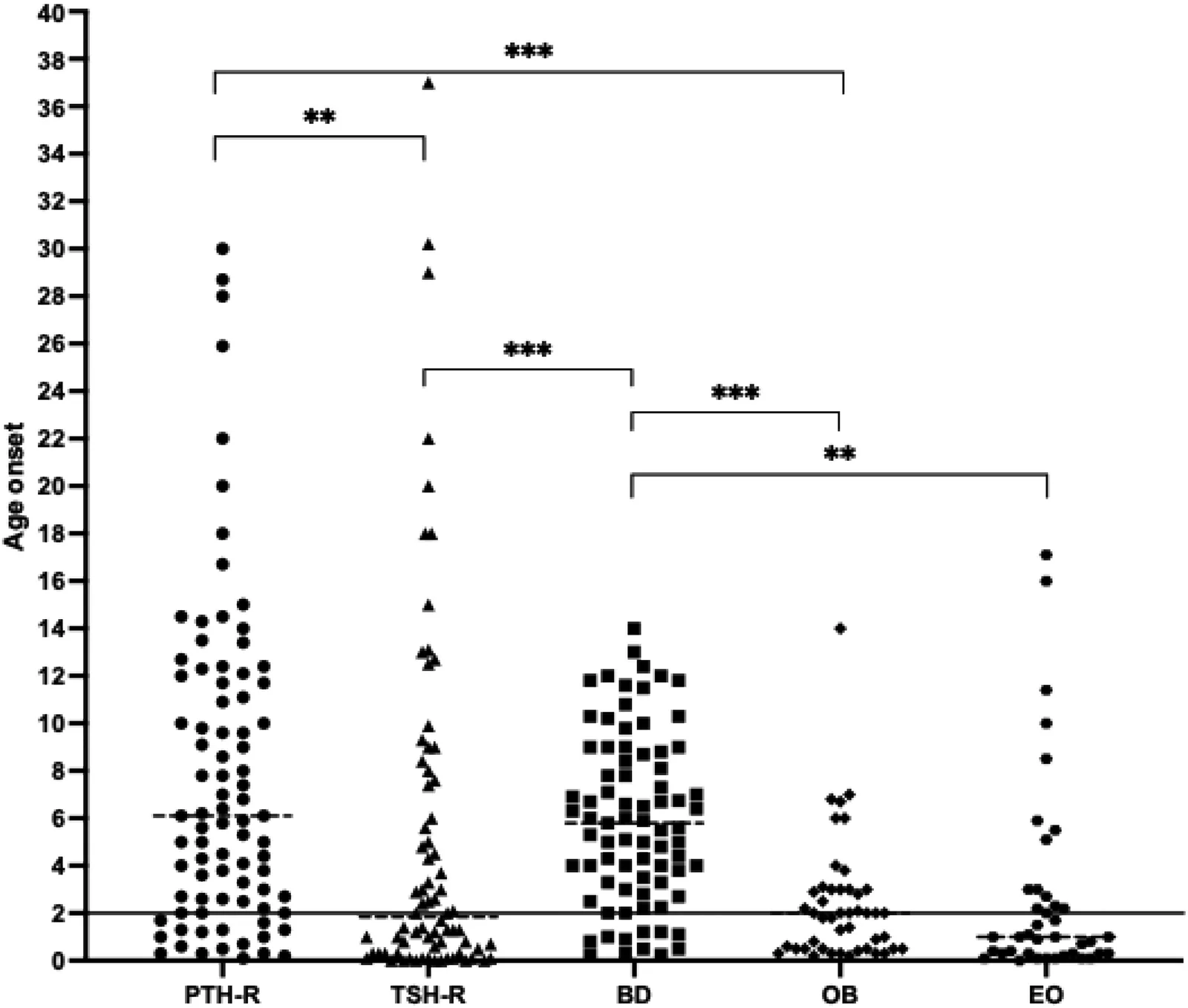
Dot plot showing the distribution of the age of onset or the age at first detection (on the y-axis) of the different clinical and biochemical features (on the x-axis) of iPPSD/PHP (all patients included): PTH-R, TSH-R, BD, OB, EO. Abbreviations: BD, brachydactyly; EO, ectopic ossifications; OB, obesity; PHP, pseudohypoparathyroidism; PTH-R, parathyroid hormone resistance; TSH-R, thyrotropin resistance. Significant differences are indicated: **P less than .05; ***P < .005.

As for the age of onset of the main clinical and biochemical features (Fig. 1, Table 1), PTH resistance was first detected at a median age of 6.1 years (IQR 2.5-11.8 years, mean 7.8 ± 6.1 years) and median PTH ULN values were 2 (IQR 1.3-4). Conversely, the age of onset of obesity (median 2 years, IQR 0.5-3.0 years, mean 2.4 ± 2.6 years) and the age at which TSH resistance was first detected (median 1.85 years, IQR 0.3-7.7 years, mean 5.2 ± 7.7 years; median TSH at diagnosis 8.5 mIU/l, IQR 6.8-12.2 mIU/l) were significantly lower (*P* = .005 and *P* = .01, respectively).

**Table 1:** Percentage distribution, age of onset, and age at first detection of the main clinical and hormonal features across iPPSD/pseudohypoparathyroidism subtypes

	Total	iPPSD1 (*PTHR1* mutation)	iPPSD2 (*GNAS* mutation)	iPPSD3 (*GNAS* methylation defect in promoter)	iPPSD4 (*PRKAR1A* mutation)	iPPSD5 (*PDE4D* mutation)	iPPSDx (unknown mutation)
No. of patients	117	3	62	21	11	5	15
Sex, F/M	67/50	2/1	32/30	11/10	8/3	4/1	10/5
Median age at diagnosis (Q1-Q3), y	5.9 (1.8-11.0)	10.3 (10.1-12.3)	2.3 (1.0-5.0)	12.2 (7.0-16.7)	7.0 (2.7-9.5)	8.0 (5.0-11.6)	11.3 (8.7-11.8)
PTH resistance, %	88 (75)	2 (67)	46 (74)	21 (100)	11 (100)	1 (20)	7 (47)
Median age at PTH resistance diagnosis (Q1-Q3), y	6.1 (2.5-11.8)	13.0 (12.3-13.6)	2.7 (1.4-6.0)	12.0 (5.9-14.0)	8.6 (3.7-11.3)	3.6	13.4 (11.3-14.5)
TSH resistance, %	77 (66)	1 (33)	51 (82)	13 (62)	11 (100)	0 (0)	1 (7)
Median age at TSH resistance diagnosis (Q1-Q3), y	1.85 (0.3-7.7)	0.7	1.2 (0.1-2.6)	13.1 (9.3-18.0)	7.6 (3.6-9.5)		NA
Brachydactyly, %	85 (73)	2 (67)	51 (82)	4 (19)	11 (100)	5 (100)	12 (80)
Median age at brachydactyly onset (Q1-Q3), y	5.8 (3.3-8.8)	6.5 (5.8-7.3)	4.9 (2.6-7.3)	6.7 (6.5-9.5)	5.0 (3.1-7.0)	5.0 (4.0-6.0)	10.3 (8.1-11.7)
Ectopic ossifications, %	40 (34)	0 (0)	39 (63)	0 (0)	0 (0)	0 (0)	1 (7)
Median age at ectopic ossifications onset (Q1-Q3), y	1.0 (0.25-2.75)		1.0 (0.3-2.5)				NA
Obesity, %	49 (42)	1 (33)	38 (61)	6 (26)	0 (0)	3 (60)	1 (7)
Median age at obesity onset (Q1-Q3), y	2.0 (0.5-3.0)	1.8	2.0 (0.6-3.0)	0.4 (0.3-0.5)		6.7 (4.3-6.8)	NA

Abbreviations: F, female; iPPSDs, inactivating PTH/PTHrP signaling disorders; M, male; NA, not available, missing data; PTH, parathyroid hormone; Q, quartile; TSH, thyrotropin.

Similarly, BD was first described at a median age of 5.8 years (IQR 3.3-8.8 years, mean 5.9 ± 3.6 years), which is significantly later than the age of onset of TSH resistance (*P* = .00) and obesity (*P* = .002).

In contrast, no statistically significant difference was detected between the age of onset of PTH resistance and BD (*P* = .9) and between the age of onset of TSH resistance and obesity (*P* = .4).

EOs are unique and specific to patients affected by iPPSD2/PHP1A/POH; this has somehow impaired statistical significance of some tests (ie, the age of onset of PTH resistance and EOs are not significantly different, *P* = ns; while the age of onset of BD is significantly higher than the age of onset of EOs; *P* = .04). In fact, almost 65% of patients in our entire cohort have never developed EOs.

**Figure 2:**
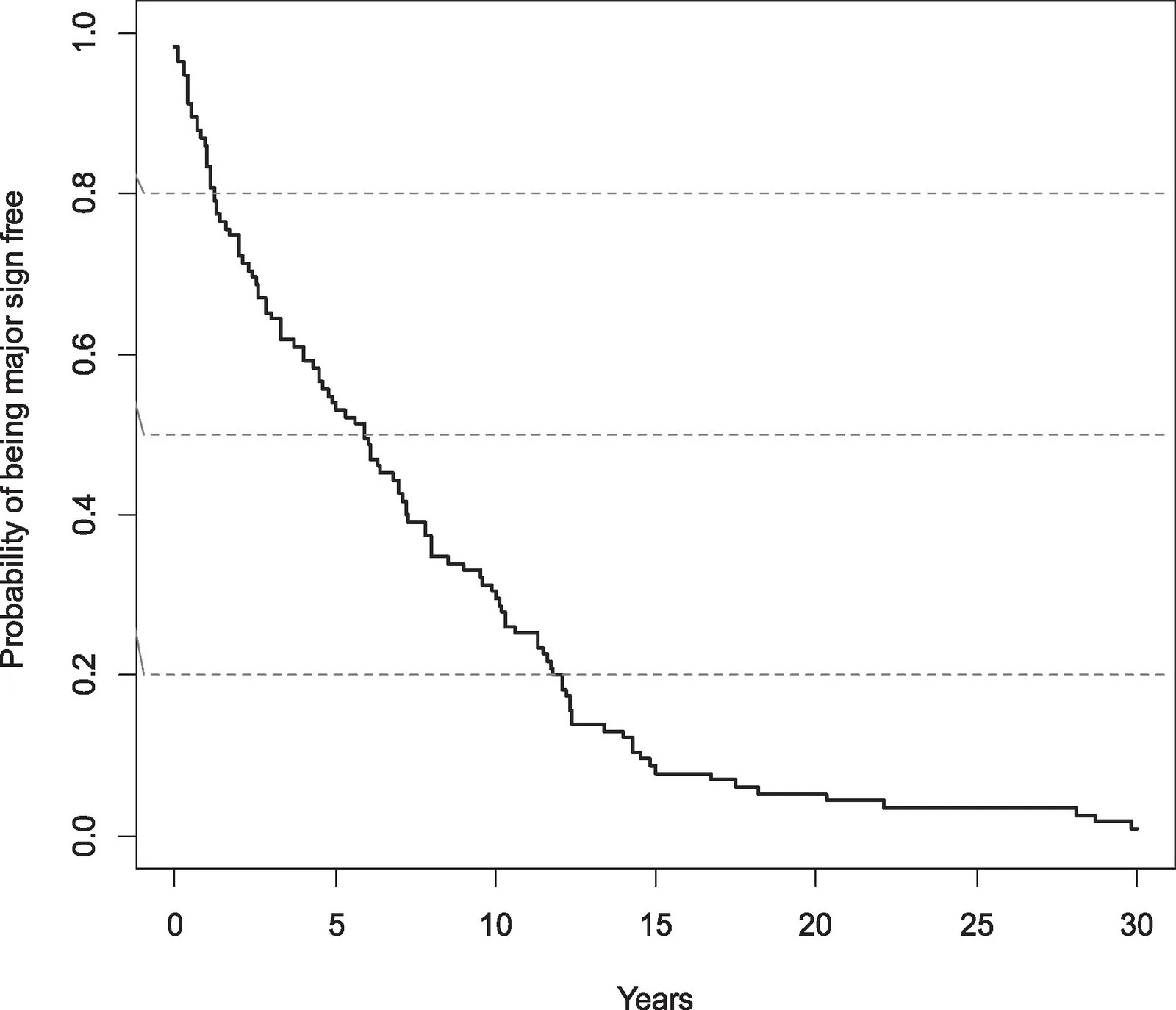
Kaplan-Meier curve showing the percentage of patients presenting with major criteria (that is probability of being major criteria free) overtime (years, on the x-axis).

Therefore, considering major criteria for diagnosis, the median age at which the first sign is detected is 4.2 years (IQR 1.0-9.0 years); at 2 years 64% of patients did not present with any major sign while at 10 years only 20% of patients have not yet developed any major criteria (Fig. 2).

**Figure 3:**
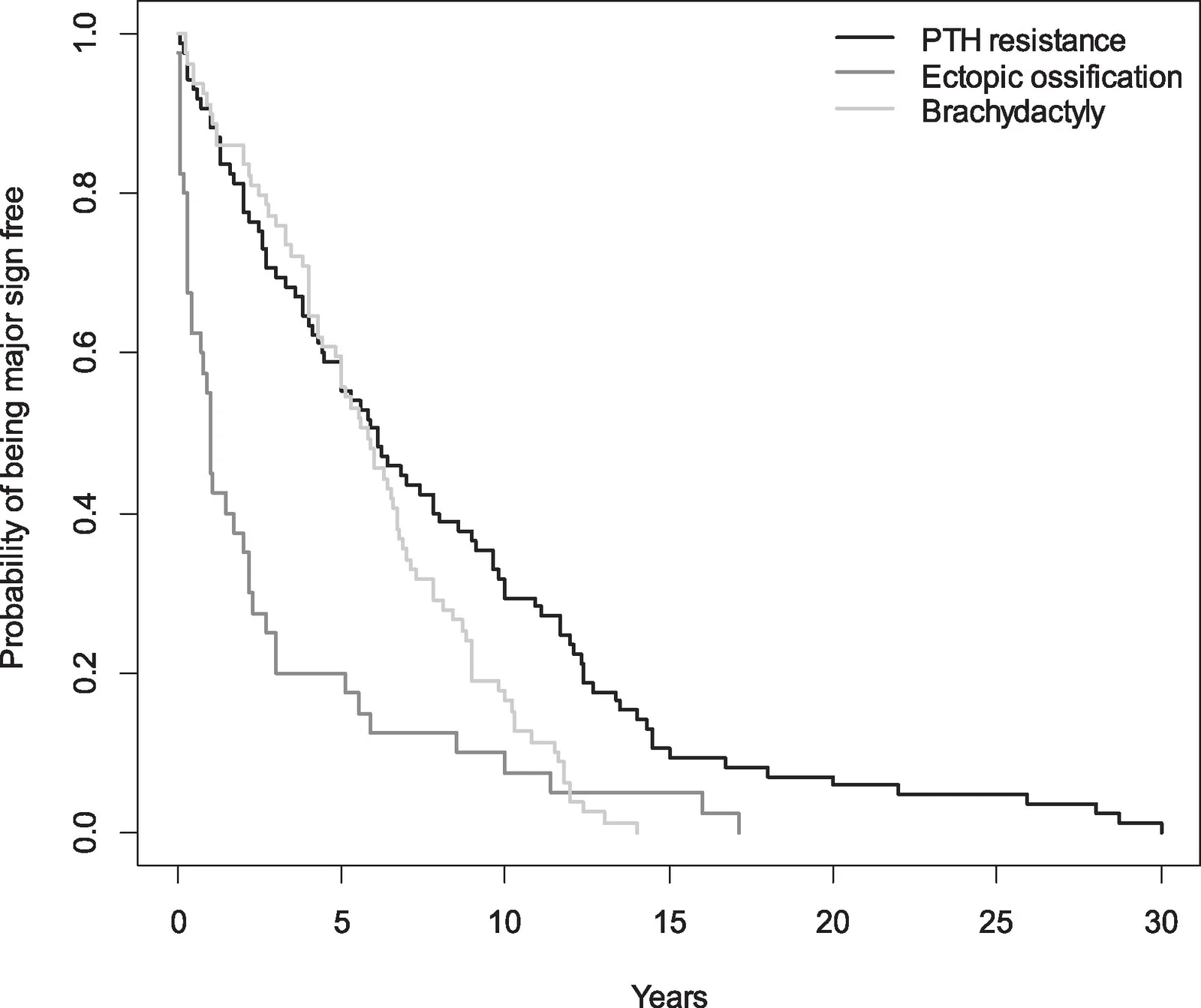
Kaplan-Meier curves showing percentage of patients presenting with PTH resistance, ectopic ossifications and brachydactyly (that is probability of being major criteria free) overtime (years, on the x-axis). Three different curves for each major criterion are shown.

In detail, 80% of patients developed EOs at 3 years, 50% of patients presented with BD at 5.6 years, and 50% received a diagnosis of PTH resistance at 6 years (Fig. 3).

Table 1 shows the differences among iPPSD/PHP subtypes regarding diagnostic age and age of onset and detection of main clinical and hormonal features. Noteworthy is that the diagnosis and the detection of main features occur earlier in iPPSD2/PHP1A than in other iPPSD/PHP subtypes.

Moreover, comparing diagnostic age and age of onset of clinical and hormonal features between patients with inherited mutations and patients with no family history of iPPSD/PHP, we found that, as expected, patients who inherited the disease were diagnosed earlier, and hormonal resistances were also detected earlier than in patients with de novo mutations. On the other hand, clinical features, including BD, obesity, and EOs, did not differ as regards to age of onset between patients with inherited mutations and patients with de novo mutations (Table 2).

**Table 2:** Comparison between patients with inherited diseases and patients with de novo mutations regarding age of diagnosis and age of onset and detection of clinical and hormonal features, respectively

Variable	Inherited mutation (n = 42)	De Novo mutation (n = 41)	*P*
Median diagnostic age (Q1-Q3), y	2.55 (0.5-5.5)	7.8 (2.0-12.2)	**.002**
Median age of TSH resistance onset (Q1-Q3), y	0.8 (0.1-2.4)	7.4 (0.8-15.0)	**.002**
Mean age of PTH resistance onset (SD), y	4.4 (3.5)	9.2 (8.1)	**.003**
Median age of brachydactyly onset (Q1-Q3), y	5.0 (2.2-7.6)	5.5 (3.5-9.0)	.28
Median age of obesity onset (Q1-Q3), y	2.0 (0.6-2.9)	2.1 (0.4-6.0)	.40
Median age of ectopic ossifications onset (Q1-Q3), y	1.7 (0.3-5.5)	0.4 (0.2-1.0)	.13

*P* values < .05 highlighted in bold.

Abbreviations: PTH, parathyroid hormone; Q, quartile; TSH, thyrotropin.

In detail, patients without a family history of iPPSD/PHP accounted for 41 out of 117 cases (35%). Among them, 11 patients (27%) were diagnosed before age 2 years (mean age 1.2 ± 0.5 years). In most of these early-diagnosed patients (7/11, 64%), the first clinical feature that prompted clinical evaluation was the presence of EOs, while 2 cases (18%) were brought to medical attention because of TSH abnormalities. In the remaining patients the first signs were EOs associated with TSH resistance and BD, respectively (Supplementary Table S2) (20).

As for diagnostic criteria, we studied the distribution of both major and minor criteria in patients before age 2 years. In 34.5% of patients TSH resistance was diagnosed before age 2 years and 36.1% of patients developed obesity or overweight before that age, while 22.2%, 16.7%, and 11.8% of patients were diagnosed with EOs, PTH resistance, or BD before age 2 years, respectively.

**Figure 4:**
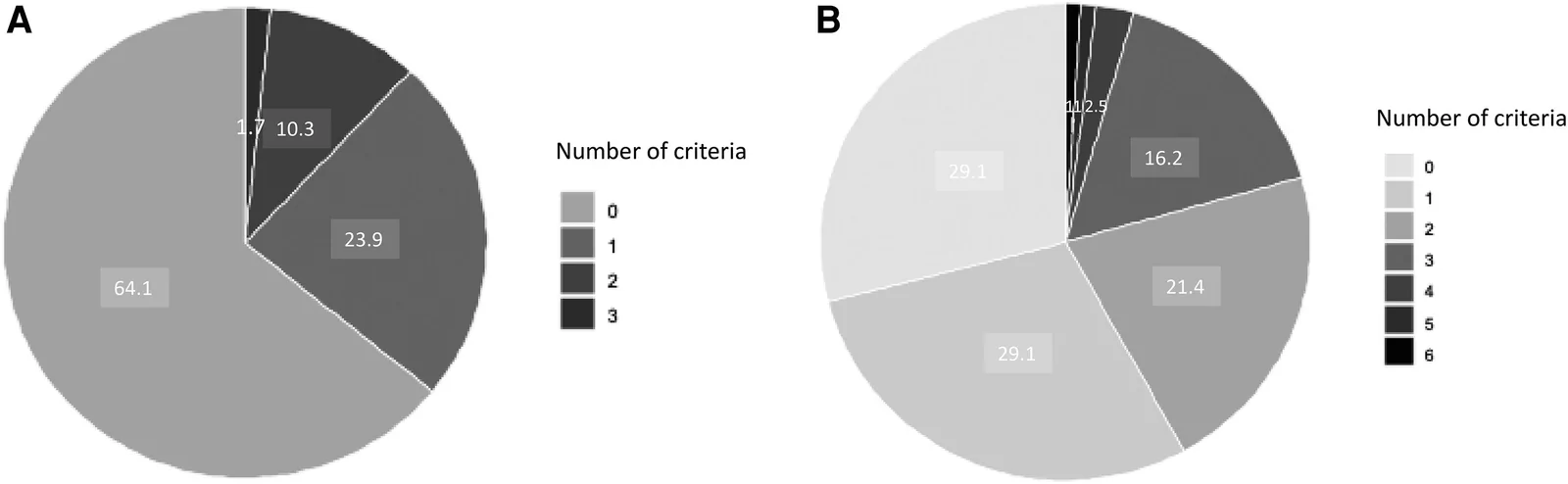
Pie charts showing the distribution of the number of major (A) and minor (B) diagnostic criteria observed before age 2 years.

Fig. 4 illustrates the number of A, major and B, minor criteria present before age 2 years.

Minor criteria were more represented than major criteria in this population (*P* = .002). Indeed, in 64% of patients before age 2 years, no major criteria were described, while 71% had already developed at least 1 minor criterion.

Minor criteria considered in our study were intrauterine growth restriction (IUGR), cognitive impairment, TSH resistance, other hormonal resistances, short stature, and obesity/overweight.

Therefore, we evaluated the prevalence of the various associations of major and minor criteria before age 2 years (Table 3), with the final goal of proposing new diagnostic criteria more useful and appropriate for early infancy. In 20% of patients TSH resistance and obesity/overweight have been simultaneously detected before age 2 years, showing a similar prevalence to main major criteria in that age group (PTH resistance and EOs, 16.7% and 22.2%, respectively).

**Table 3:** Distribution of clinical and biochemical features before age 2 years

	N	%
OB/OW	39/108	36.1
TSH resistance	39/113	34.5
EOs	26/117	22.2
NC	22/100	22
PTH resistance	19/114	16.7
BD	13/110	11.8
OB/OW + TSH resistance	23/114	20
OB/OW + TSH resistance + EOs	10/115	9
OB/OW + TSH resistance + NC	10/112	9
BD + OB/OW + NC	4/92	4.3
BD + TSH resistance + NC	4/95	4.2
BD + PTH resistance	5/116	4
BD + EOs	4/110	3.6
BD + TSH resistance + OB/OW	3/101	3

Abbreviations: BD, brachydactyly; EOs, ectopic ossifications; NC, neurocognitive impairment; OB/OW, obesity/overweight; PTH, parathyroid hormone; TSH, thyrotropin.

BD is less specific than the other major criteria and should be combined with at least 1 major or 2 minor criteria to trigger the diagnosis of iPPSD/PHP as previously suggested (1, 22). In our cohort, BD and PTH resistance or EOs were simultaneously detected in only 3% to 4% of patients before age 2 years, which is considerably lower than the 20% observed in patients with TSH resistance and obesity/overweight.

Even comparing the concomitant prevalence of BD and 2 minor criteria (ie, TSH resistance, NC, or obesity/overweight), we found a remarkable difference with respect to the prevalence of TSH resistance associated with early-onset obesity/overweight.

## Discussion

The term *pseudohypoparathyroidism (PHP)*, newly referred to as inactivating PTH-PTHrP signaling disorder (iPPSD), includes a group of rare diseases whose exact prevalence is unknown and estimated to be 1 of 295 000 in Japan (23), 1 of 150 000 in Italy (Orphanet ID No. 12935), 1.1 of 100 000 in Denmark (24), and probably close to 1 of 20 000 in the United States (25). The diagnostic work-up leading to the diagnosis of iPPSD/PHP starts from clinical and biochemical features that may vary depending on the age of the patient, family history, and mode of inheritance. The median age at diagnosis in our study cohort was 6 years (mean 7 years), similar to the age of onset of the iPPSD/PHP major features described in the literature, that is, PTH resistance and BD (22). In particular, as for patients with iPPSD2/PHP1A, PTH resistance, the central hallmark of these diseases, develops gradually over time and overt symptomatic hypocalcemia is present in most patients by age 7 to 8 years (5, 12, 13, 26). In this scenario, an early diagnosis of iPPSD/PHP can thus be challenging, and a diagnostic delay is commonly described.

As previously reported by Kayemba-Kay's et al (16), iPPSD2/PHP1A seems to have a bimodal clinical and biological presentation pattern. In fact, in their cohort features such as lunar face, subclinical hypothyroidism, subcutaneous ossifications, and rapid weight gain/obesity were the most prevalent signs in toddlers (age <2 years), while moderate intellectual disability, BD, hypocalcemic seizures, short stature, and overt TSH resistance predominated in children older than 2 years (16).

Thus, iPPSD/PHP can have an atypical pattern of onset and development during early infancy.

We include all these disorders under the common definition of “PTH resistance syndromes” and, as such, we expect the occurrence of PTH resistance to raise the first diagnostic suspicion in almost all cases. Other clinical features are suggestive of iPPSD/PHP and present minimal overlap with other clinical conditions; for this reason they have been included among major diagnostic criteria: EOs and BD (1). Nevertheless, the clinical picture is more complex and major criteria are often absent, particularly during early infancy.

As for PTH resistance, patients often do not present with hypocalcemia or elevated PTH levels until after a few years of life (12). To explain this latency, knockout mouse models have been studied and showed that PTH resistance develops with age, despite the presence of the molecular defect since conception, leading to the hypothesis that there is a gradual silencing of the paternal Gsα in the renal proximal tubule (13). In accordance with these observations, PTH resistance in our cohort was first detected at a median age of 6.1 years (IQR 2.5-11.8 years) and was described in only 16.7% of patients before age 2 years. Only 6 patients (5%) in our entire cohort were diagnosed after a hypocalcemic seizure. Some authors speculate that vitamin D and/or calcium supplementation could also play a role in delaying the occurrence of hypocalcemia in toddlers (16), but unfortunately we do not have data about early vitamin D and calcium supplementation in our entire cohort.

Among the major diagnostic criteria, BD is another specific although not early clinical sign. It becomes clearly recognizable during late childhood or prepuberty (14), except in patients diagnosed with acrodysostosis, in which the shortening of metacarpals and metatarsals is usually observed in early infancy (6). Similarly to PTH resistance, the median age at first detection of BD in our cohort was 5.8 years (IQR 3.3-8.8 years) and BD was described before age 2 years in only 11.8% of patients.

Conversely, EOs are specific signs of inactivating mutations of *GNAS* exon 1 to 13 and they usually appear earlier during infancy, even at birth in some cases in the form of osteoma cutis (5, 11). Riepe et al (27) previously reported the case of a newborn presenting with hypothyroidism and subcutaneous ossifications who was subsequently diagnosed with iPPSD2/PHP1A, and concluded that calcinosis cutis in an infant or child should lead to the suspicion of iPPSD/PHP. In line with this case, EOs in our series were described at a median age of 1 year (IQR 0.25-2.75 years), and we observed that these are the only early manifestations among the major diagnostic criteria.

Indeed, there are some reports in the literature on early clinical and biochemical features in iPPSD/PHP, supporting the hypothesis that major criteria are not so accurate for diagnosis during early infancy (11-13, 28). These are either single case reports or small case series. Our data confirm and better elucidate this aspect, highlighting that the age of onset of PTH resistance and BD is significantly greater than the age of onset of TSH resistance, obesity, and EOs. We can therefore speculate that PTH resistance/BD and TSH resistance/early obesity represent 2 pairs of clinical features that show a strong age dependence.

TSH resistance has often been reported as one of the first biochemical signs of iPPSD/PHP (29-31) even at birth, being in some cases misdiagnosed as congenital hypothyroidism (17, 32, 33). Thus, if some authors suggest screening for iPPSD/PHP all infants presenting with concomitant hypothyroidism and EOs (27), others recommend considering PTH resistance syndromes when there is simultaneous detection of congenital hypothyroidism and hyperphosphatemia, even if Albright Hereditary Osteodystrophy (AHO) is missing (32).

Early-onset obesity is another minor criterion variably associated with some iPPSD/PHP subtypes (15, 34-36), and together with TSH resistance it could be considered as one of the earliest manifestations suggesting the diagnosis. Recently, a large cohort of patients with severe early-onset obesity was screened for loss-of-function mutations of *GNAS,* revealing an unexpectedly high prevalence (22/2548, 1%) (37). Interestingly, also in these patients TSH resistance was an early feature, while PTH resistance and overt hypocalcemia emerged over time, confirming the findings of the previous series. The authors seem to suggest that screening for *GNAS* children with early onset of severe obesity may allow a reduction in diagnostic delay, similarly to that previously suggested by Kayemba-Kay's and colleagues in their series of 10 patients (16).

In our study cohort, TSH resistance was detected at a median age of 1.85 years (IQR 0.3-7.7 years) and it was already diagnosed in 34.5% of patients before age 2 years. Similarly, the median age of onset of obesity was 2 years (IQR 0.5-3.0 years) and 36.1% of patients presented with obesity or overweight before age 2 years.

Trying to dissect the best association of diagnostic criteria suitable for early infancy, TSH resistance and early-onset obesity have shown a higher prevalence in children before age 2 years (20%), in comparison to the association of 2 major criteria such as PTH resistance and BD, that were present in only 4% of children before that age, or to the association of BD and 2 minor criteria as suggested by current guidelines.

**Figure 5:**
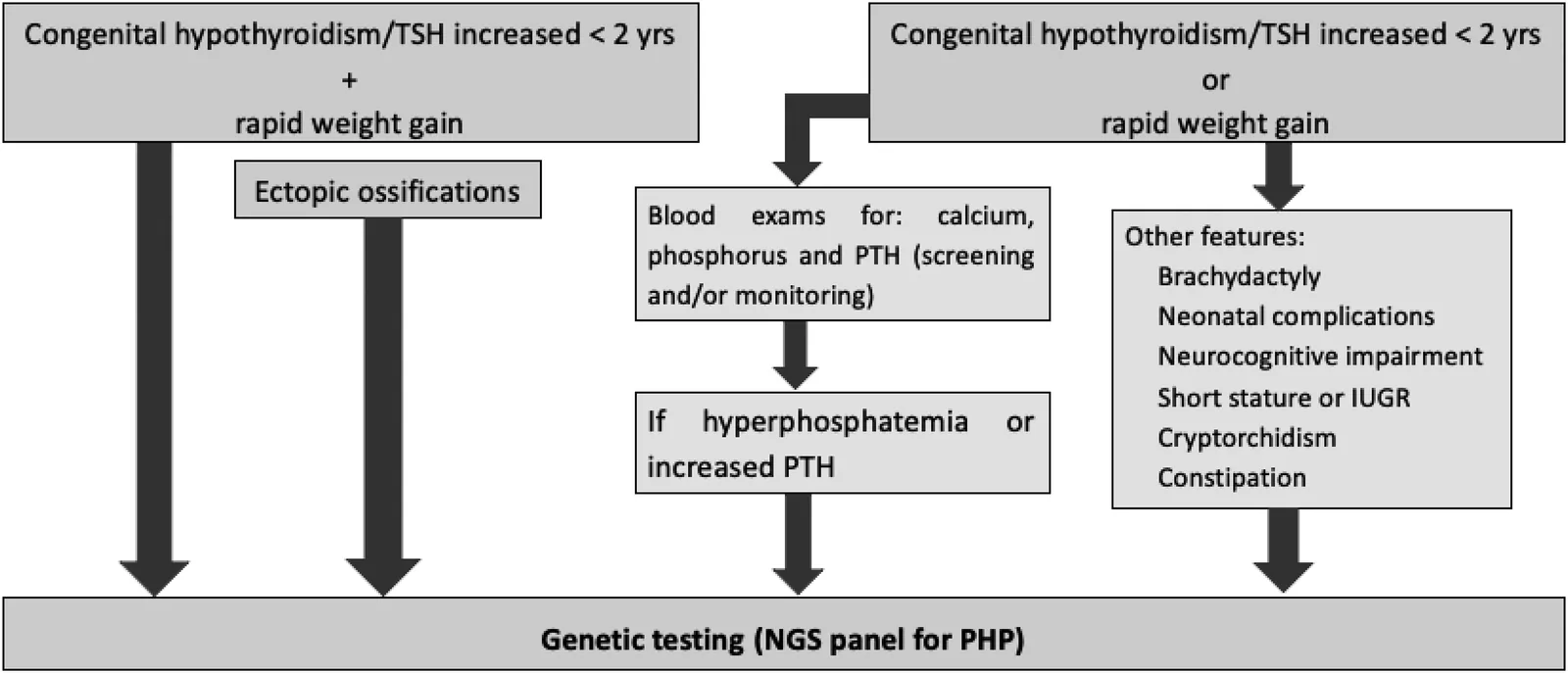
Proposed flowchart for diagnosis of iPPSD/PHP patients during early infancy. TSH resistance combined with rapid weight gain before age 2 years, or the presence of ectopic ossifications, may warrant genetic testing (NGS panel for iPPSD/PHP), while isolated TSH resistance or isolated rapid weight gain before age 2 years should be associated with other suggestive features before recommending genetic testing.

Fig. 5 proposes a diagnostic flowchart for early infancy. In line with our findings, we suggest the importance of suspecting PTH resistance syndromes in children presenting with early-onset obesity and increased TSH levels. Specifically, in our proposed diagnostic flowchart, the coexistence of TSH resistance and rapid weight gain before age 2 years represents a potential early warning sign that may lead to genetic testing after careful clinical evaluation. Conversely, the presence of only one of these two findings should be interpreted in the context of other suggestive features, such as additional hormonal resistances, BD, elevated PTH levels, NC, etc.

Unfortunately, statistical analysis stratified by iPPSD/PHP subtype could not be performed, due to the small size of some cohorts (ie, iPPSD4, iPPSD5, or iPPSD1). Notably, iPPSD2/PHP1A represents a more complex clinical entity, characterized by both hormonal resistances and the constellation of physical features defined as AHO, which includes a round face, a stocky habitus with short stature, BD, and EOs (5). This could partly explain why diagnostic age, as well as the age of onset of main clinical or biochemical features, is lower in this subgroup of patients compared to others (see Table 1).

However, when comparing patients with inherited mutations to those with de novo mutations, no differences in age of onset of clinical features were observed, whereas biochemical abnormalities were usually detected earlier in patients with inherited disease (see Table 2). This finding is rather expected, as patients with a known family history of iPPSD/PHP are typically screened and followed up earlier, highlighting the need for further studies exploring the natural disease history in newborns of affected adults.

### Conclusions

The major features of iPPSD/PHP usually appear in late childhood with consequent common diagnostic delay. Nevertheless, a few complications and clinical or biochemical features are already present in the neonatal period or early infancy. Our study confirms the previous series reporting a bimodal age-dependent clinical scenario and points out that current diagnostic criteria may not be accurate to diagnose the disease in toddlers. Reducing the diagnostic delay may have important consequences. First, it may prevent the most severe clinical complications, such as hypocalcemia-related seizures. Moreover, accounting for the important effect of iPPSD2/PHP1A and acrodysostosis on statural growth, an earlier diagnosis could allow us to better monitor height and growth rate to introduce recombinant human GH (rhGH) replacement therapy during the limited time window for a potentially effective therapy (38). Finally, reducing diagnostic delay could also improve both genetic counseling for families planning to have another child and multidisciplinary management of the affected children. In our cohort, the most represented clinical and biochemical features in the first years of life were TSH resistance and early-onset obesity/overweight, currently included among minor diagnostic criteria, pointing out that diagnostic criteria should be reviewed and differentiated according to age.

### Limitations

We acknowledge that this study is limited by the retrospective nature of the analysis. For this reason, we could not retrieve all data on the age of onset of clinical and biochemical features. Further prospective studies are needed to better clarify the natural early-infancy history of iPPSD/PHP, particularly enrolling all newborns who inherited the disease from known adult patients. Thus, our conclusions are to be understood as recommendations and a basis for future research. Although being the largest cohort of patients reported in the literature to date, the relatively small number of participants limits the conclusions that can be drawn for each iPPSD/PHP subtype. However, this study establishes the need for revising diagnostic criteria for early infancy, as a bimodal diagnostic approach for children and adults is probably requested.

## Data Availability

All data generated or analyzed during this study are included in this published article or in the supplemental information; the data underlying this article are available in Supplementary material for “Clinical Picture of Early Infancy PTH Resistance Syndromes: Is It Time to Improve Diagnostic Criteria?” at https://doi.org/10.13130/RD_UNIMI/Y4TQJQ.

## Abbreviations

AHO: Albright Hereditary Osteodystrophy
BD: brachydactyly
BMI: body mass index
cAMP: cyclic adenosine monophosphate
EOs: ectopic ossifications
GH: growth hormone
Gsα: α-subunit of the stimulatory G protein
IOTF: International Obesity Task Force
iPPSDs: inactivating PTH/PTHrP signaling disorders
IQR: interquartile range
NC: neurocognitive impairment
PHP: pseudohypoparathyroidism
PTH: parathyroid hormone
PTHrP: parathyroid hormone–related protein
TSH: thyrotropin
ULN: upper limit of normal
